# Persistent Insulin Autoimmune Syndrome in a Caucasian Male in the Absence of Triggers

**DOI:** 10.7759/cureus.58270

**Published:** 2024-04-14

**Authors:** Dinesh Edem, Jhansi Maradana, Priyanka Majety, Mc Anto Antony, Lakshmi Menon

**Affiliations:** 1 Endocrinology, Diabetes and Metabolism, University of Arkansas for Medical Sciences, Little Rock, USA; 2 Endocrinology, Diabetes and Metabolism, Mass General Brigham Wentworth-Douglas Hospital, Dover, USA; 3 Endocrinology, Diabetes and Metabolism, Virginia Commonwealth University, Richmond, USA; 4 Endocrinology, Diabetes and Metabolism, Medical University of South Carolina, Anderson, USA

**Keywords:** low carb diet, insulin autoimmune syndrome, postprandial hypoglycemia, hirata's disease, autoimmune hypoglycemia, high serum insulin, insulin antibodies

## Abstract

Insulin autoimmune syndrome (IAS) or Hirata disease is a rare condition presenting as recurrent hypoglycemia, and associated with elevated insulin levels in the presence of insulin autoantibodies (IAAs) in patients who were never exposed to exogenous insulin and with no evidence of pancreatic abnormalities. IAS is much more frequent in East Asians, especially the Japanese population, compared to the lower incidence in Caucasians. However, it can be associated with other autoimmune diseases or drug use like methimazole and alpha-lipoic acid (ALA).

We report a case of a 47-year-old Caucasian male presenting with a 12-month history of worsening episodes of fasting and post-prandial hypoglycemia associated with symptoms of dizziness, tremors, palpitations, and unconsciousness associated with hypoglycemia. Symptoms resolved with the administration of carbohydrate-containing foods, establishing Whipple’s triad. At an outside facility, he had initial labs that showed elevated insulin levels (141 µU/ml) with normal glucose, C-peptide, and proinsulin levels, but there was no availability of an IAA lab assay. Given his symptoms, severity, and frequency of hypoglycemia, he was admitted to the hospital for a 72-hour fast, which showed the lowest glucose level of 64 mg/dl with inappropriately high insulin of 22.2 µU/ml, low C-peptide of 0.57 ng/ml, and undetectable proinsulin of <1.6 pmol/L, but with IAA being >50 U/ml (0.0-0.4 U/ml). He was treated with intensive dietary counseling with a low-carbohydrate diet and prednisone 20 mg twice daily initially. Additionally, he could not tolerate octreotide, diazoxide, and acarbose due to side effects. He is currently on prednisone 10 mg daily and nifedipine with no further hypoglycemic episodes, but still has a high IAA of >50 U/ml and serum insulin levels of 70-112 µU/ml.

Our case highlights the importance of recognizing hypoglycemia and checking for IAA levels as first-line diagnostic tests, in the absence of which there could be a delay in diagnosis and leading to unnecessary lab and imaging testing. Our case is unique since it happened in a Caucasian without any prior exposure to a triggering factor and has not undergone self-remission yet, which happens in most of IAS cases.

## Introduction

Hypoglycemia is a condition in which plasma glucose is low enough to cause adrenergic and/or neuroglycopenic symptoms and signs. Hypoglycemia is confirmed by Whipple’s triad, where all three criteria must be met, namely, symptoms or signs consistent with hypoglycemia, a low plasma glucose concentration, and resolution of symptoms or signs after plasma glucose is raised [1]. Insulin autoimmune syndrome (IAS), or Hirata’s disease, is a rare condition presenting as recurrent spontaneous hyperinsulinemic hypoglycemia, which was described by Hirata et al. in 1970 [2,3]. IAS is associated with markedly elevated insulin levels in the presence of insulin autoantibodies (IAAs) in patients who were never exposed to exogenous insulin and had no evidence of pathologic pancreatic abnormalities [4]. Hypoglycemia is most likely post-prandial and/or fasting and could also occur after physical exertion [4,5].

IAS appears to be a form of type VII hypersensitivity that can be associated with certain human leukocyte antigens (HLAs), especially HLA DRB1*0406, which is more frequent in East Asians; hence, IAS was the third most common cause of hypoglycemia in Japan [6]. However, IAS in Caucasians appears to be associated with HLA DRB1*0403, which has a much lower incidence overall [3,5]. IAS can either be idiopathic or can be associated with other autoimmune diseases such as Graves’ disease, systemic lupus erythematosus, rheumatoid arthritis, or monoclonal gammopathy or with certain drugs such as methimazole, carbimazole, and alpha lipoid acid (ALA) [3,5]. Due to its rare occurrence in Caucasians and the lack of IAA assays in some labs, IAS could often go undiagnosed for a significant period of time, with several unnecessary lab and imaging tests, resulting in significant morbidity for the patients. IAS is mostly a self-remitting disease but can be challenging if it becomes persistent, with treatment being stopping the offending drugs, low-carbohydrate meal plan, glucocorticoids, alpha-glucosidase inhibitors, immunosuppressant or even plasma exchange [3-5,7,8]. There is no clear consensus on treatment guidelines, except for case reports or review papers.

We present a case of persistent IAS with delayed diagnosis in a Caucasian male without self-remission, being treated with dietary changes, glucocorticoids, and nifedipine.

## Case presentation

A 47-year-old male with no history of diabetes presented to our endocrine clinic for evaluation of fasting and post-prandial hypoglycemia with episodes of dizziness, tremors, palpitations, and unconsciousness associated with low blood glucose (BG) for the past 12 months. He was doing well until one year ago, when he noted feeling dizzy and lightheaded while driving, leading to an episode of disordered driving, which improved after eating a carbohydrate-containing food. Five months later, he had another episode where he lost control of the car, went completely quiet, and spoke incoherent sentences, which improved after eating. Over the past six months, he has had worsening and increasingly frequent episodes of dizziness, tremors, palpitations, speaking in incoherent sentences, and unconsciousness associated with a low plasma BG of <70 mg/dl. Symptoms resolved with the administration of carbohydrate-containing foods, fulfilling Whipple's triad. He has noted night-time fasting and post-meal symptoms. He endorsed 20 lbs. of weight gain in the last year due to eating foods constantly to keep his glucose in the normal range.

His past medical history was significant for obesity, and he was not on any medications. He never had gastrointestinal or bariatric surgery. Vital signs were normal, except for a BMI of 32. Apart from abdominal adiposity, the physical examination was unremarkable. Fasting outpatient workup revealed insulin levels of 86.1-141 µU/ml (2.6-24.9 µU/ml), plasma glucose of 90-140 mg/dl (70-105 mg/dl), C-peptide of 1.3-4.1 ng/ml (1.1-4.4 ng/ml), and proinsulin of 1.6-2.5 pmol/L (0.0-10.9 pmol/L). Other causes of hypoglycemia, like surreptitious diabetic medications, exogenous insulin, adrenal insufficiency, infections, severe illness, thyroid, liver, and kidney dysfunction, were ruled out. Given his endogenous hyperinsulinemia, he had an abdominal ultrasound, computed tomography (CT), and magnetic resonance imaging (MRI) of the abdomen, all of which did not reveal a pancreatic mass.

**Table 1:** Initial outpatient labs before the endocrinology visit. Elevated insulin levels with normal glucose levels suggest insulin binding with its autoantibodies. BG: blood glucose, HbA1c: hemoglobin A1c, TSH: thyroid-stimulating hormone, ACTH: adrenocorticotropic hormone.

Labs	Results
HbA1c (4.8%–6.4%)	5.4
Fasting BG (70–105 mg/dl)	97
Fasting insulin (2.6–24.9 µU/ml)	86
Fasting C-peptide (1.1–4.4 ng/ml)	1.3
Proinsulin (0.0–10.9 pmol/L)	1.6
Sulfonylurea screen	Negative
TSH (0.34–5.60 µIU/ml)	0.91
Cortisol (6.2–19.4 µg/dl)	10.1
ACTH (7.2–63.3 pg/ml)	23

Given his symptoms, their severity, and frequency, he was admitted to the hospital for a 72-hour fast. Lab work during a hypoglycemic episode showed plasma glucose of 64 mg/dl, with inappropriately high insulin of 22.2 µU/ml, low C-peptide of 0.57 ng/ml, with undetectable proinsulin of <1.6 pmol/L, negative sulfonylurea and meglitinide screen, normal ACTH, cortisol and insulin-like growth factor-1 (IGF-1). Beta-hydroxybutyrate was elevated at 3.69 mmol/L (<0.27 mmol/L), and the response to the glucagon stimulation test was <25 mg/dl in 30 minutes, indicative of depletion of glycogen reserves. His insulin auto-antibodies were high at >50 U/ml (0.0-0.4 U/ml), suggesting insulin autoimmune syndrome (IAS). Continuous glucose monitor (CGM) sensor BG checks showed a clear relationship between high-glycemic foods with resultant glucose spikes with subsequent hypoglycemia two to four hours later. 

**Table 2:** Labs at the beginning of the 72-hour fasting and before the end of the fast. The patient didn’t have any hypoglycemic symptoms with a glucose of 63; hence, it was an inconclusive test. Elevated insulin autoantibody results returned post-discharge, at which point he was started on treatment for IAS. IAS: insulin autoimmune syndrome.

72-hour fasting labs	Day 1	Day 3
Blood glucose (70–105 mg/dl)	115 (H)	63 (L)
Insulin (2.6–24.9 µU/ml)	92.3 (H)	20.4
C-peptide (1.1–4.4 ng/ml)	2.22	0.52 (L)
Proinsulin (0.0–10.9 pmol/L)	<1.6	<1.6
Beta-hydroxybutyrate (≤0.27 mmol/L)	0.92 (H)	3.69 (H)
Insulin antibody (0.0–0.4 U/ml)	>50.0 (H)	>50.0 (H)

Dietary counseling was provided to lower carbohydrates and increase protein, fats, and fiber-containing foods without much improvement. He was started on prednisone 20 mg twice daily, and octreotide 200 mcg three times daily, which could not be continued due to GI side effects. He was discharged home on prednisone and increasing doses of diazoxide with much improvement in BGs and symptoms. He had significant edema and weight gain with diazoxide 50 mg three times daily, resulting in stopping the medication. He was then switched to acarbose, which worked well but caused extensive rashes with the subsequent discontinuation of medication. He was then changed to prednisone 10 mg twice daily and nifedipine 60 mg ER daily, which he has tolerated for more than six months. Fifteen months after the diagnosis, he has been maintained on a low-carb diet, prednisone 10 mg once daily, and nifedipine 60 mg ER daily. He has lost 20 lbs. in the past year, now with a BMI of 29. He has significant and frequent hypoglycemic episodes whenever he stops taking prednisone or nifedipine. He continues to have insulin antibodies >50 U/ml and insulin levels ranging from 70 to 112 µU/ml; however, on his current regimen, his hypoglycemic episodes, as reported in the CGM, are very rare but tolerable.

## Discussion

Our patient’s case is unique, as there was a delay in the diagnosis of his insulin autoimmune syndrome (IAS), and he had no history of prior exposure to any triggering factor and no spontaneous self-remission.

IAS is a rare cause of spontaneous hyperinsulinemic hypoglycemia, initially described by Hirata et al. in 1970, which is characterized by markedly elevated insulin levels due to the presence of insulin autoantibodies (IAA), especially in patients with no prior exogenous insulin use or any pancreatic abnormality [4]. IAS can manifest either as post-prandial and/or fasting hypoglycemia, where insulin is bound to its autoantibodies, which have a high binding capacity to insulin and low affinity in nature. Post-prandially, carbohydrates in the meals result in pancreatic insulin secretion, but the insulin is strongly bound to the IAA, causing insulin-IAA complexes, resulting in post-prandial hyperglycemia and even higher insulin release. When the binding capacity is exceeded, the insulin dissociates from IAA and now causes significant post-prandial hypoglycemia two to five hours after a meal [9,10]. CGM studies have shown IAS to be associated with alternating post-prandial hyperglycemia followed by hypoglycemia [7,11]. Symptoms are initially adrenergic/autonomic (tremors, anxiety, hunger, and sweating), followed by neuroglycopenia (irritability, behavior change, confusion, loss of consciousness, or seizures). Weight gain has also been associated with elevated insulin levels and the need to eat constantly to avoid post-prandial hypoglycemia [1].

IAS has been classified as either idiopathic or drug-related, especially sulfhydryl-containing medications, namely methimazole [12], carbimazole, sulfonylureas, or ALA [3,13]. Sulfhydryl-containing medications bind and reduce disulfide bonds linking two insulin chains, making them more immunogenic to the antigen-presenting cells, resulting in the development of IAA [5]. IAS has also been associated with other autoimmune disorders in at least half of the cases in the Asian population. Oest et al. and Wammer et al. mention two distinct subtypes of IAS in East Asian and Caucasian populations [5,14]. It is postulated that IAS in East Asians is more drug-induced, has higher rates of self-remission, and is more associated with HLA subtype DRB1*0406, whereas the IAAs are polyclonal [15]. In contrast, IAS in Caucasians are more idiopathic with lesser rates of self-remission, association with HLA subtype DRB1*0403 and the IAA’s being more monoclonal [3]. IAS is still a rare disease entity, except in Japan, where it’s the third most common cause of hypoglycemia; however, worldwide prevalence is increasing given higher rates of offending drug/supplement use, greater clinical awareness, and routine checking of IAA’s [4].

The first step in diagnosing the etiology of hypoglycemia would be to confirm hypoglycemia by documenting Whipple’s triad. In patients who fulfill Whipple’s triad and do not have an obvious etiology for hypoglycemia, the next step is to proceed to the 72-hour fast to diagnose hyperinsulinemic hypoglycemia. Diagnosis of hypoglycemia includes insulinoma vs nesidioblastosis vs post-bariatric hypoglycemia vs exogenous insulin use vs use of hypoglycemic drugs. Oftentimes, cases of IAS are mislabeled as insulinoma, leading to inappropriate and expensive lab and diagnostic tests, which mostly occur due to a lack of clinician awareness of this disease entity. The gold standard for diagnosis of IAS involves elevated levels of insulin and IAA’s. In the absence of exogenous insulin use, another giveaway is the presence of markedly elevated insulin levels compared to C-peptide levels, resulting in an insulin: C-peptide ratio of >1, which is typically <1 in insulinoma and other hyperinsulinemic states [3,9].

There are no definitive guidelines or consensus on the therapies for IAS. Most of the therapies are from case reports or case series with no comparison studies [3,4]. IAS is usually a self-remitting disease in three to six months, with offending drug withdrawal being the best initial step [9]. Management should be aimed at preventing the post-prandial glucose spike with a low-carbohydrate meal plan [16], with frequent small meals low in simple sugars resulting in lower rates of post-prandial hypoglycemia. Along similar lines, alpha-glucosidase inhibitors like acarbose [9] or voglibose reduce the breakdown of disaccharides to monosaccharides, preventing post-prandial hyperglycemia and hypoglycemia. Tapering doses of glucocorticoids have been successfully used to help with the remission or control of hypoglycemia. Other immunosuppressants, like azathioprine [17] and rituximab [7], have been used in various case reports with good success. Other treatments like octreotide [9], diazoxide [18], and partial pancreatectomy have been less commonly used. Plasmapheresis [8] has been successfully used as a last resort in emergencies. Calcium channel blockers (CCBs) like nifedipine and verapamil have been shown to reduce insulin secretion through inhibition of voltage-gated calcium channels on pancreatic β-cells [19]. There have been case reports [20] that have shown the effectiveness of nifedipine in cases of hyperinsulinemic hypoglycemia, especially in infants and post-bariatric hypoglycemia.

Our patient had a prolonged taper of glucocorticoids with a recurrence of hypoglycemic symptoms whenever his prednisone or nifedipine was tapered. He continues to have elevated IAA but significantly reduced hypoglycemic episodes on prednisone and nifedipine.

## Conclusions

Hypoglycemia can cause significant morbidity and a structured diagnostic approach should be undertaken to discern its causes. IAS should be considered in the differential diagnosis of hyperinsulinemic hypoglycemia, which can be diagnosed by measuring IAAs in the setting of elevated insulin levels. Given the easier availability of medications and supplements that can contribute to the development of IAS, it is imperative to be aware of this disease entity, to prevent further unnecessary diagnostic testing. There is no definitive pharmacotherapy and further research is needed. First-line treatment involves stopping the offending drug and initiating low-carbohydrate meal plans, steroids, or other immunosuppressants. However, this is a mostly self-remitting disease with a good prognosis.
